# Isolation and characterization of head and neck cancer-derived peritumoral and cancer-associated fibroblasts

**DOI:** 10.3389/fonc.2022.984138

**Published:** 2022-12-05

**Authors:** Jiefu Zhou, Sabina Schwenk-Zieger, Gisela Kranz, Christoph Walz, Frederik Klauschen, Sharduli Dhawan, Martin Canis, Olivier Gires, Frank Haubner, Philipp Baumeister, Vera Kohlbauer

**Affiliations:** ^1^ Department of Otorhinolaryngology, Head and Neck Surgery, Grosshadern Medical Center, Ludwig-Maximilians-University (LMU), Munich, Germany; ^2^ Institute of Pathology, Faculty of Medicine, Ludwig-Maximilians-University (LMU) Munich, Munich, Germany; ^3^ Clinical Cooperation Group “Personalized Radiotherapy in Head and Neck Cancer”, Helmholtz Zentrum München, German Research Center for Environmental Health GmbH, Neuherberg, Germany

**Keywords:** head and neck squamous cell carcinoma, cancer-associated fibroblasts, peritumoral fibroblasts, tumor microenvironment, angiogenesis

## Abstract

**Introduction:**

Head and neck squamous cell carcinomas (HNSCC) are characterized by strong cellular and molecular heterogeneity and treatment resistance entailing poor survival. Besides cell-intrinsic properties, carcinoma cells receive important cues from non-malignant cells within the tumor microenvironment (TME). Cancer-associated fibroblasts (CAFs) are a major component of the TME that impact on the molecular make-up of malignant cells and have a decisive function in tumor progression. However, the potential functionality of fibroblasts within tumor-adjacent, macroscopically normal tissue remains poorly explored.

**Methods:**

Here, we isolated primary peritumoral fibroblasts (PtFs) from macroscopically normal tissue in vicinity of primary human papillomavirus-negative and -positive oropharyngeal HNSCC and compared their phenotype and functionality with matched CAFs (n = 5 pairs) and with human oral fibroblasts (hOFs).

**Results:**

Expression patterns of CD90, CD73, CD105, smooth muscle actin, Vimentin, and S100A4 were comparable in PtFs, CAFs, and hOFs. Cell proliferation and doubling times of CAFs and PtFs were heterogeneous across patients (n =2 PtF>CAF; n = 1 CAF>PtF; n = 2 CAF=PtF) and reflected inferior growth than hOFs. Furthermore, PtFs displayed an reduced heterogeneity in cell size compared to matched CAFs, which were characterized by the presence of single large cells. Overall, conditioned supernatants from CAFs had more frequently growth-promoting effects on a panel of carcinoma cell lines of the upper aerodigestive tract carcinoma cell lines (Cal27, Cal33, FaDu, and Kyse30), whereas significant differences in migration-inducing effects demonstrated a higher potential of PtFs. Except for Kyse30, CAFs were significantly superior to hOFs in promoting proliferation, while PtFs induced stronger migration than hOFs in all carcinoma lines tested. Analysis of soluble factors demonstrated significantly increased VEGF-A production in CAFs (except in pat.8), and significantly increased PDGF-BB production in PtFs of two patients. Tube formation assays confirmed a significantly enhanced angiogenic potential of conditioned supernatants from CAFs compared to hOFs on human umbilical vascular endothelial cells (HUVECs) *in vitro*.

**Discussion:**

Hence, matched CAFs and PtFs present in HNSCC patients are heterogeneous in their proliferation-, migration-, and angiogenesis-promoting capacity. Despite this heterogeneity, CAFs induced stronger carcinoma cell proliferation and HUVEC tube formation overall, whereas PtFs promoted migration of tumor cells more strongly.

## Introduction

ENT (ear, nose, and throat) cancers arise in the lips, oral cavity, paranasal sinuses, pharynx, salivary glands, larynx, and trachea. Most of these malignancies are head and neck squamous cell carcinomas (HNSCCs) that are treated with a combination of surgery, chemotherapy, and radiotherapy. However, treatment resistance and local recurrence remain significant problems in the therapy efficacy (1). Consequently, HNSCCs have a poor overall 5-year survival below 50% especially in the case of classical HNSCC that are commonly induced by tobacco and alcohol abuse (2). In contrast, HNSCC associated with a chronic infection with high-risk sub-types of the human Papillomavirus have better survival prognosis (3).

Improving treatment efficacy of HNSCCs and applying patient-tailored treatment requires a deeper understanding of the underlying mechanisms that confer therapy resistance and metastatic potential (4). In this respect, non-malignant cells of the tumor microenvironment (TME) are crucial determinants of tumor initiation, progression, and therapy resistance (5, 6). The TME is a multicellular ecosystem containing mesenchymal and endothelial cells, including cells of hematopoietic origin, arranged in the extracellular matrix (ECM), which interact with cancer cells and regulate tumorigenesis. The most prominent non-immune cell type contained in the TME of HNSCC is cancer-associated fibroblasts (CAFs). These non-malignant cells of the TME are crucial determinants of tumor progression (7, 8), and assume critical roles in promoting epithelial-to-mesenchymal transition (EMT), angiogenesis, and evasion of tumor cells from the host’s immunological response (9, 10). In HNSCCs, CAFs have the potential to accelerate the progression by secreting growth factors, altering the ECM, vasculature, and increasing therapy resistance, enabled by symbiotic communication through secreted factors (11, 12). In late stage HNSCC, up to 80% of the tumor volume frequently consists of CAFs (13). On the other hand, there are fewer reports suggesting a tumor-suppressive role of CAFs *via* the I kappa B kinase/NF-κB pathway, which leads to reduction of hepatocyte growth factor (HGF) secretion and reduction of tumor size and metastasis (14).

In contrast to normal fibroblasts, the precise origin of CAFs remains unclear and a matter of debate. They may either originate from inactivated residential “normal” fibroblasts (15, 16), from a differentiation of mesenchymal progenitors that have been attracted from their localization within the bone marrow, or from transdifferentiated tumor-resident mesenchymal cells (17). CAFs may be activated by different growth factors and cytokines, such as fibroblast growth factor (FGF), transforming growth factor-β (TGF-β), platelet-derived growth factor (PDGF), receptor tyrosine kinase (RTK) signaling, tumor necrosis factor (TNF), or by reactive oxygen species (ROS), among others (8, 18).

Generally, CAFs can be identified by the expression of selected markers, such as α-smooth muscle actin (α-SMA), Vimentin, S100A4, CD90, CD73 and CD105 (19–21). However, more recent research suggests that different CAF subpopulations exist, which have distinct molecular characteristics and functions (22, 23). Single cell RNA sequencing revealed the existence of eight CAF subtypes preferentially present in HNSCC compared with normal tissue, with each cluster exhibiting differing transcriptomic profiles and selected subclusters being associated with poor overall survival (24). Another study found 14 different subtypes of CAFs in advanced stage HNSCC tumors pre- and post-treatment with αPD-L1 therapy, nivolumab, whereby one subtype was able to predict patient response to nivolumab therapy (25).

Despite these emerging differences between CAFs, little is known about frequencies and characteristics of fibroblasts found in macroscopically normal tissue in the periphery of HNSCC. This aspect is important since field cancerization, which is considered a source of frequent recurrences and secondary tumors in HNSCC, affects such apparently healthy tissue areas. It remains unclear how fibroblasts in the vicinity of primary HNSCCs compare to CAFs in terms of their molecular characteristics and function. Therefore, this study was conducted to isolate and perform a comparative characterization of matched pairs of CAFs and peritumoral fibroblasts (PtFs) from primary resected HNSCC before adjuvant treatment.

## Materials and methods

### Tissue collection

Patients consented to collection of oropharyngeal squamous cell carcinoma (OPSCC) tissue during routine surgery by punch biopsy from peripheral areas of the tumor to exclude the presence of necrotic tissue. Microscopically normal mucosa was obtained approximately one cm beyond clear resection margins after tumor removal, intraoperatively proven by frozen section analysis. All samples originate from patients treated at the Department of Otorhinolaryngology, Head and Neck Surgery, University Hospital, LMU Munich, Germany (Ethics number 18-446). As per guidelines of the eighth edition of the TNM staging, p16 expression served as marker for the association of OPSCC with chronic HPV infection. Additionally, p16-positive tumors were subjected to HPV-typing using the consensus GP5+ and GP6+ primers detecting the late HPV gene L1 on the VisionArray HPV Chip 1.0 (ZytoVision, Bremerhafen, Germany).

### Isolation, cell culture, and supernatant collection of CAFs and PtFs

CAFs and PtFs were isolated from matched pairs of tumor tissue and microscopically normal mucosa of OPSCC patients (n=5 patients termed Pat.3, Pat.4, Pat.6, Pat.7 and Pat.8), respectively. To isolate CAFs and PtFs from tumor and mucosa samples, each tissue was washed three times in five mL PBS and subsequently transferred into one mL Fibroblast Growth Medium (FGM, Promo Cell C-23020, Heidelberg, Germany) supplemented with ten mg/mL Collagenase P (#11213857001, Roche, Mannheim, Germany) for two hours at 37°C. Thereafter, cell pellets were centrifuged (1000 rpm for five mins), supernatant was discarded, and the cell pellets were resuspended into 12 mL FGM with supplements [20% Fetal Calf Serum (FCS), one ng/mL Basic Fibroblast Growth Factor (BFGF), five µg/mL Insulin (Promo Cell), 1% Penicillin/Streptomycin (#11548876, Gibco, Paisley, UK), and 1% Gentamicin (A2712, Biochrom, Berlin, Germany)] and maintained at 37°C and 5% CO_2_. For supernatant collection, 2x10^5^ cells of CAFs or PtFs were seeded in a 75 cm^2^ flask in 12 mL supplemented FGM for four days. Thereafter, fresh medium was added, and the conditioned supernatant was collected 72 hours later. All supernatants were frozen at -80°C until further use.

Human oral fibroblasts (hOFs) were purchased from Innoprot, Derio, Spain (P10868) and were cultured under the same conditions as CAFs and PtFs in FGM with 20% Fetal Calf Serum (FCS), 1 ng/mL Basic Fibroblast Growth Factor (BFGF), five µg/mL Insulin (Promo Cell), 1% Penicillin/Streptomycin (#11548876, Gibco, Paisley, UK), and 1% Gentamicin (A2712, Biochrom, Berlin, Germany).

HNSCC and esophageal permanent cancer cell lines Cal27, Cal33 (oral cavity), FaDu (hypopharynx), and Kyse30 (esophageal) were purchased from ATCC (ATCC, Manassas, VA, USA) and were regularly controlled for mycoplasm contamination. All cell lines were cultured in DMEM or RPMI, 10% FCS, 1% penicillin/streptomycin, 5% CO_2_ atmosphere at 37°C.

### PtF and CAF proliferation assay

Cell proliferation of PtFs and CAFs was measured using the Colorimetric Cell Viability Kit I (WST-8, PK-CA705-CK04, PromoCell GmbH, Heidelberg, Germany) according to the manufacturer’s instructions. In brief, PtFs and CAFs were seeded at a density of 500 cells/well in 96-well plates, and cell viability was measured on days one, three, and six (D1, D3, D6) at 450nm. Absorption values on D3 and D6 were normalized to D1.

### PtF and CAF doubling time

To measure doubling times of PtFs and CAFs at passages one to seven, 1x10^5^ PtFs/CAFs were seeded in 25 cm^2^ flasks and cultured for four days. Cells were then trypsinized and counted by trypan blue staining at every passage. Doubling times of matched PtFs and CAFs were calculated using the formula:


$$Doubling\;time=\text{duration}*\frac{\text{log}(2)}{\log(final\;concentration)-(initial\;concentration)}$$

### Quantification of cell size

PtFs or CAFs were seeded on glass slides (2-3x10^4^ cells/slide) for 24 hours and pictures were taken with an Olympus BX43 microscope (Olympus, Hamburg, Germany). Cell size was quantified by ImageJ software after manually tracing cell borders. Each slide was divided into equally sized quadrants and four to five non-overlapping cells were randomly selected from each quadrant to quantify cell size. Two to three slides were analyzed accordingly for each sample, thus achieving a total of 30 cell/sample.

### Flow cytometry

For flow cytometry-based detection of cell surface antigens, hOFs, CAFs, and PtFs from passages one to two were used. 50,000 to 100,000 cells were incubated with conjugated primary antibodies in PBS for one hour at 4°C, and, after washing, were resuspended in one mL of ice-cold PBS with 3% FCS for analysis. Measurement was performed in a Cytoflex flow cytometry device (Beckman Coulter, Krefeld, Germany) and analyzed using the CytExpert software (Beckman Coulter). All antibodies and isotpype controls were purchased from BioLegend, California, USA and are described in **Supplementary Table 1**.

### Immunocytochemistry staining and quantification of antigen expression

CAFs, PtFs, and hOFs were seeded on coverslips at a density of 10,000 cells/slide and cultured in supplemented FGM for 24 hours. Staining was performed according to a modified protocol from “IHC/ICC Protocol Guide” (R&D Systems, Minneapolis, USA). PtFs and CAFs were stained for the expression of three markers expressed in fibroblasts (rabbit monoclonal; alpha smooth muscle actin (α-SMA) (#M0851, Agilent Dako, Santa Clara, CA, USA); Vimentin (#ab92547, Abcam, Cambridge, UK); S100A4 (#ab197896, Abcam, Cambridge, UK) and three markers absent in fibroblasts (rabbit monoclonal; E-cadherin (#3195, Cell Signaling, Danvers, USA); Pan-cytokeratin (#501085A, Invitrogen, Carlsbad, CA, USA); CD31 (#M823, Agilent Dako, Santa Clara, CA, USA) at a dilution of 1:200. Images were acquired with an Olympus BX43 microscope (Olympus, Hamburg, Germany). All antibodies are listed in **Supplementary Table 1**. ICC intensity scores were calculated as the product of the percentage of antigen-positive cells and antigen expression intensity ranging from 0-3 (providing an ICC score range of 0-300). Quantification was performed by two independent experienced scorers in a sample-blinded manner.

### Cancer cell proliferation

To evaluate the effect of PtF and CAF supernatants on tumor cell proliferation, 1x10^5^ cancer cells (Cal27, Cal33, Kyse30, FaDu) were seeded in 6-well plates for 24 hours. Thereafter, cells were washed once with PBS and, subsequently, cultured in 1.3 mL conditioned supernatant from PtF and CAF cultures, or 1.3 mL control media including: supernatant of carcinoma cells cultured in FGM medium for 72 hours (supernatant negative control); supernatant of carcinoma cells cultured in DMEM/RPMI 1640 with 10% FCS (positive control) or without FCS (negative control) for 72 hours. Alternatively, carcinoma cell lines were cultured in 1.3 mL hOF-derived supernatant under identical conditions. Cell numbers of carcinoma cells/well were counted after 72 hours incubation with conditioned media/controls.

### Enzyme-linked immunosorbent assay

Secretion of vascular endothelial growth factor A (VEGF-A), platelet-derived growth factor BB (PDGF-BB), transforming growth factor β2 (TGF-β2), and brain-derived neurotrophic factor (BDNF) was measured using the Human VEGF-A ELISA Kit (PromoCell GmbH, Heidelberg, Germany); PDGF-BB Human SimpleStep ELISA Kit (ab184860), TGF-β2 Human ELISA Kit (ab100648) and Human BDNF SimpleStep ELISA Kit (ab212166) from Abcam, Cambridge, UK. All assays were performed according to the manufacturer’s protocol.

### Cancer cell migration assay

Cancer cell migration of carcinoma cells in the presence of PtF/CAF/hOF conditioned medium was quantified according to protocol using the QCM™ 24-well colorimetric cell migration assay kit (ECM-508, Sigma-Aldrich, Germany). Briefly, 3x10^5^ carcinoma cells were plated in 24-well trans-well plates in 300 µL serum-free medium for 24 hours. The lower chamber was filled with 500 µL with either of the following culture supernatants: conditioned supernatants of PtFs, CAFs or hOFs; supernatants of carcinoma cells cultured in DMEM or RPMI 1640 for 72 hours (negative control); serum-free DMEM or RPMI 1640 medium (blank). Carcinoma cells were incubated in the trans-well for 24 hours and cells that migrated to the lower surface of the transwell were stained with 400 µL cell stain. Subsequently, 200 µL extraction buffer was added for 15 mins at room temperature and 100 µL of the dye mixture was transferred to a 96-well microtiter plate suitable for colorimetric measurement at 560 nm.

### Angiogenesis (tube formation) assay

Tube formation assay was performed as described previously (26). Briefly, 10 µL Matrigel Matrix GFR (Corning, Fisher Scientific, Schwerte, Germany) was added into a µ-plate angiogenesis 96-well (Ibidi, Gräfelfing, Germany). Human umbilical vein endothelial cells (HUVECs, PromoCell, Heidelberg, Germany) in passage two to five were grown to 80% confluence in Endothelial Cell Growth Medium (ECGM, PromoCell, Heidelberg, Germany), trypsinized, and cell pellet was resuspended in either PtF/CAF/hOFs supernatants from patients #3, #4 and #8, fresh supplemented ECGM, or HUVEC-conditioned FGM supernatant. The cells were then plated into the µ-plate 96-well at a density of 1x10^4^ cells/well and cultured for eight hours. Photographs of each well were taken with a Leica DMi8 microscope at five-fold magnification in phase contrast. At least five technical replicates of each sample were taken, the result of three independent experiments are displayed. The images were analyzed using ImageJ’s “Angiogenesis Analyzer” module. Tubes were determined and quantified as a measure of angiogenic potential.

### Statistical analysis

Student’s T-test and one-way ANOVA was used for statistical analysis using the Prism 9 software. A p-value of ≤ 0.05 was considered significant.

## Results

### Isolation of matched pairs of CAFs and PtFs

In the process of surgical removal of primary OPSCCs, punch biopsies were obtained from the primary tumor and microscopically normal mucosa. The latter samples were taken beyond resection margins that were deemed clear after intraoperative frozen section analysis by pathologists. Clinical parameters of the OPSCC are summarized in **Table 1**. All patients except Patient #4 suffered from advanced OPSCC with nodal involvement. The HPV-status was assessed using a recommended two-tier method including p16 accumulation measured by immunohistochemistry and high-risk HPV typing according to the eighth edition of the TNM classification (27). Three patients showed p16 accumulation and carried HPV high-risk strain 16 or 33. Furthermore, smoking status, extracapsular, perineural, lymphatic and vascular invasion are summarized in **Table 1**.

**Table 1:** Clinical parameters of OPSCC patients. ECE, extracapsular extension; PNI, perineural invasion; LI, lymohatic invasion; VI, vascular invasion.

Patient	TNM-status	p16/HPV DNA	Smoking status	ECE	PNI	Localization	LI	VI
#3	pT3 pN1 cM0	pos./HPV16	former	pos.	neg.	oropharynx	pos.	neg.
#4	pT2 pN0 cM0	pos./HPV33	former	neg.	neg.	oropharynx	neg.	neg.
#6	pT3 pN2b cM0	Neg.	current	neg.	pos.	oropharynx	pos.	pos.
#7	pT2 pN1 cM0	pos./HPV16	former	neg.	neg.	oropharynx	neg.	neg.
#8	pT3 pN3b cM0	Neg.	former	pos.	neg.	oropharynx	pos.	neg.

**Figure 1:**
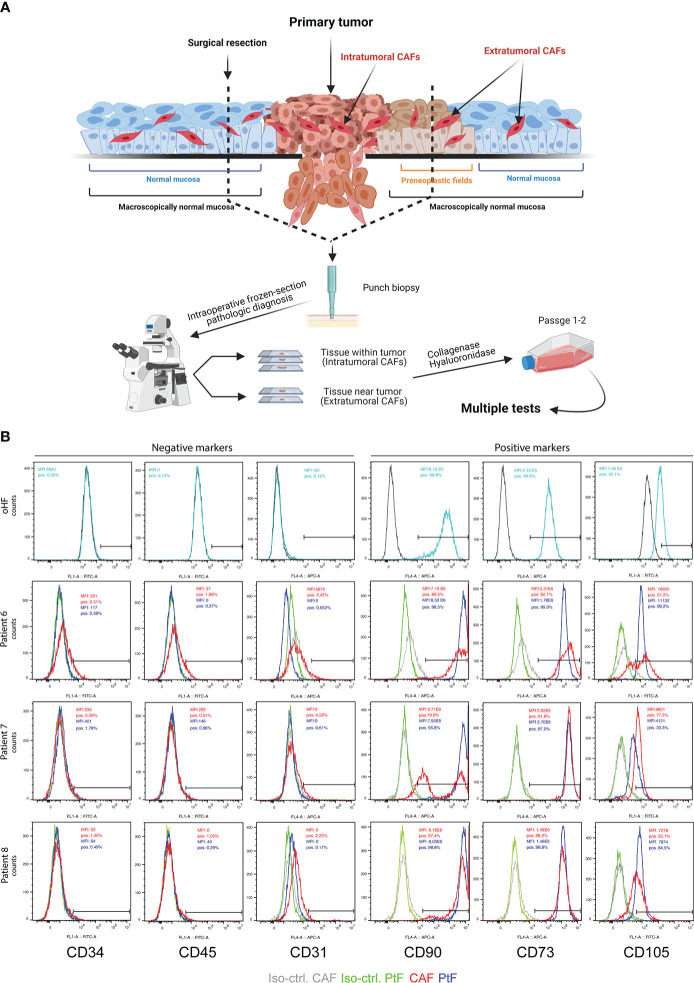
Establishment of PtFs and CAFs from OPSCC patient samples. **(A)** Schematic representation of the workflow to establish peri- and intratumoral CAFs (PtFs and CAFs, respectively). Punch biopsies were taken from areas of microscopically normal mucosa and primary OPSCC. After pathological diagnosis on frozen tissue sections during surgery, single cells were generated from punch biopsies and PtFs and CAFs were isolated. **(B)** PtFs and CAFs in passages 1-2 and hOFs were stained for the expression of fibroblast markers (CD90, CD73 and CD105) and markers for leukocytes, hematopoietic cells, and endothelial cells (CD45, CD34 and CD31, respectively). Expression levels were assessed by flow cytometry and percentage of positive cells standardized for cognate isotype controls are shown in the top right corner of the histogram for both PtFs (blue) and CAFs (red). The same markers were assessed in human oral fibroblasts (hOF) and are shown in the upper panels. **Figure 1A** was generated using BioRender und publication license number WQ242UAZXN

Fibroblasts isolated from primary tumor tissue were defined as cancer-associated fibroblasts (CAFs), whereas fibroblasts isolated from normal mucosa beyond microscopically clear resection margins were defined as peritumoral fibroblasts (PtFs). CAFs and PtFs were obtained from n = 5 matched samples of tumor and normal mucosa after treatment with collagenase and hyaluronidase. The resulting single cell suspensions were cultured in fibroblast growth medium for further characterization (**Figure 1A**).

To confirm CAFs and PtFs as fibroblasts, primary cultures in early passages one to two were assessed for the expression of fibroblast markers CD90, CD73 and CD105, and the lack of expression of markers for leukocytes (CD45), hematopoietic cells (CD34), and endothelial cells (CD31). Fibroblast markers were expressed on the cell surface of all PtFs, whereas the CAF populations were heterogeneous for CD105 and CD90 expression in patients #6 and #7, respectively. Neither CAFs nor PtFs expressed CD45, CD34, or CD31, thereby allowing the exclusion of leukocytes, hematopoietic and endothelial cells in all fibroblast cultures (**Figure 1B**). Normal human oral fibroblasts (hOF) were analyzed for the same markers. CD31, CD34, and CD45 were not detectable on hOF, whereas CD73, CD90, and CD105 were homogeneously expressed (**Figure 1B**, upper panels).

**Figure 2:**
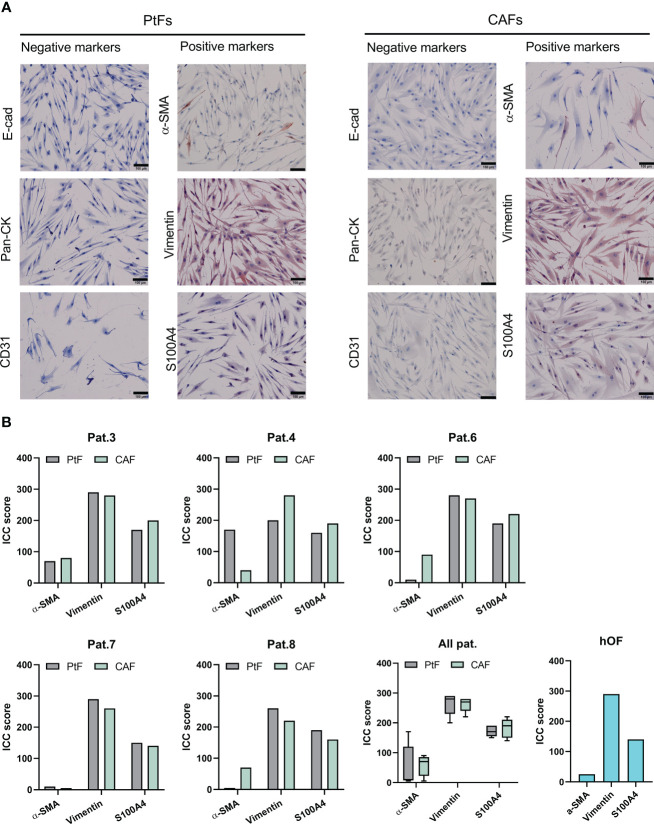
Immunocytochemistry (ICC)-based characterization of PtFs and CAFs. **(A)** Expression of epithelial markers [E-cadherin, pan-cytokeratin (Pan-CK)], endothelial marker (CD31), and the fibroblast markers (α-SMA, Vimentin, S100A4) in PtFs and CAFs was assessed through ICC staining and counter staining with hematoxylin-eosin during passages 1-2. Representative images are shown. **(B)** ICC scoring was performed on fibroblast markers of individual patients (Pat.3, Pat.4, Pat.6, Pat.7 and Pat.8), all patients combined, and human oral fibroblast (hOF; bottom right).

Results of the flow cytometry analyses were corroborated by immunocytochemistry (ICC) staining of matched CAFs and PtF pairs, and hOFs. Representative images of ICC-stained samples show that CAFs, PtFs, and hOFs have a mesenchymal, spindle-shaped cell morphology and lack the expression of epithelial markers E-cadherin and pan-cytokeratin, and the endothelial marker CD31 (**Figure 2A**, **Supplementary Figure 1**). Cell cultures were further stained for Vimentin, S1004A, and alpha-smooth muscle actin (α-SMA), a marker for a subset of activated fibroblasts termed myofibroblasts. All cells expressed Vimentin, most cells expressed S100A4, and only a subset of cells from CAFs, PtFs, and hOFs expressed α-SMA (**Figure 2A**, **Supplementary Figure 1**). Antigen expression was scored as described earlier (28), which provides a combined quantification of the proportion of antigen-expressing cells and expression intensity. ICC scores of fibroblast markers for CAFs and PtFs from individual patients as well as averaged mean ICC scores across all patients are shown in **Figure 2B**. Especially the number of α-SMA-expressing cells varied in patients #4, #6 and #8 between PtFs and CAFs, with the latter two patients exhibiting higher α-SMA expression in CAFs vs PtFs, whereas patient #4 showed the opposite pattern (**Figure 2B**). Generally, the observed expression pattern was similar to hOFs, in which Vimentin was strongly expressed, S100A4 showed an intermediate expression, and α-SMA was weakly expressed (**Figure 2B**).

In summary, CAFs and PtFs were successfully retrieved from five independent OPSCC donors from primary tumors and macroscopically normal peritumoral tissue. Both fibroblast populations expressed classical fibroblast markers, whereby CAFs of patients #6 and #7 showed heterogeneous cell populations as measured by CD90 and CD105, with some cells lacking marker expression. One cause for such differences in marker expression could be due to the presence of distinct CAF subgroups within the CAFs isolated from primary tumors, which were not accounted for in the scope of this study (24). Moreover, α-SMA expression in patients #4, #6 and #8 was not consistent suggesting different amounts of myofibroblasts and non-activated resting fibroblasts in their respective CAF and PtF populations. All CAF and PtF populations lacked expression of negative markers, thereby excluding the presence of other cell types within selected fibroblast cultures. Lastly, the observed antigen expression patterns were reminiscent of normal hOFs.

### Proliferation, doubling times, and morphology of matched CAF and PtF pairs

**Figure 3:**
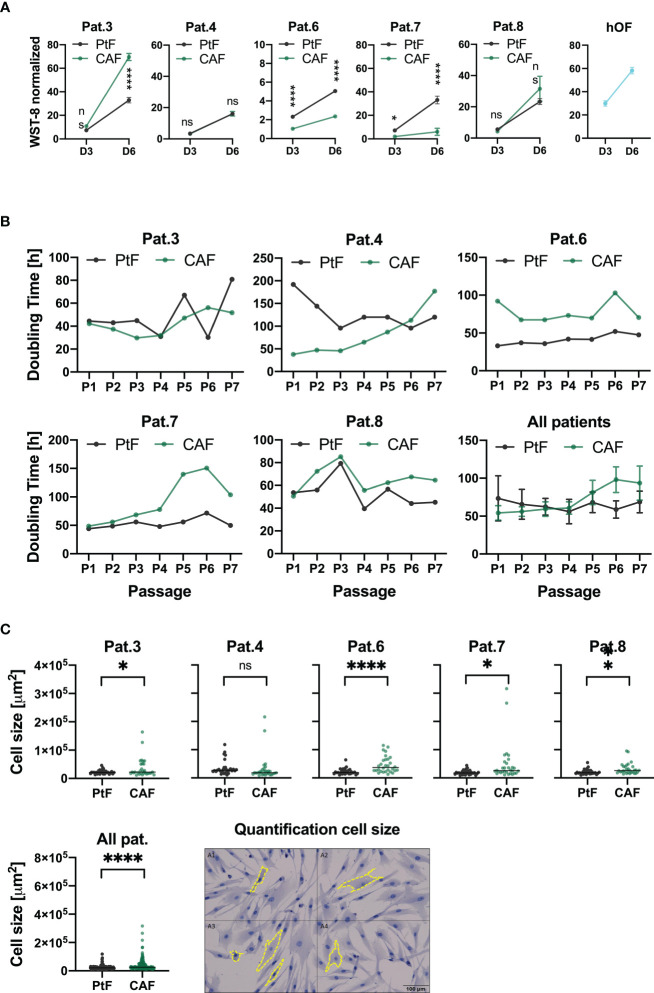
Proliferation, doubling times and cell size of PtFs and CAFs. **(A)** Cell proliferation of PtFs and CAFs of individual patients (Pat.3, Pat.4, Pat.6, Pat.7 and Pat.8), and human oral fibroblasts (hOF) were measured at D3 and D6 after seeding using a WST-8 assay. Normalized mean ± SD absorption values from n=3 independent experiments are shown. **(B)** Doubling time at each passage of matched CAFs and PtFs of all individual patients was calculated from passages 1-7. Averaged doubling times of CAFs and PtFs of all patients combined is shown in the bottom right. **(C)** Cell sizes of PtFs and CAFs were quantified using a software-based approach. Quantified cell sizes im mm^2^ (n=30 per sample) are shown. Representative image showing cell size determination (yellow dotted line) on an E-cadherin-stained sample is shown. P-values: ns > 0.05; *≤ 0.05; ** ≤0.01; **** ≤0.0001.

To compare short term proliferation of CAFs, PtFs, and hOFs, a WST-8 assay was performed on days 3 and 6 following initial seeding. Overall, differences in proliferation between PtFs and CAFs were more pronounced on D6 compared to D3 (except Pat.4 and 8 who showed no significant differences in proliferation between PtFs and CAFs on both days) (**Figure 3A**). Biggest significant differences were observed for Pat.6, and less so for Pat.7, with PtFs proliferation being higher compared to CAFs. On D6, CAFs showed higher proliferation rates for Pat.3 and lower proliferation rates for Pat.6 and Pat.7, compared to their respective PtF populations (**Figure 3A**). hOFs showed superior proliferation rates than CAFs and PtFs, except for CAFs from Pat.3 (**Figure 3A**).

Additionally, doubling times of matched pairs of PtFs and CAFs of all patients were measured between passages one to seven. Overall, PtF and CAF cultures of each patient individually showed similar trends in doubling time over the course of passages one to seven (except Pat.4), with CAFs having slightly higher doubling times that increase over time (**Figure 3B**). Doubling times of all patients combined showed that PtFs do not change over time, whereas CAFs increase their doubling times slightly.

At the morphological level, CAFs appeared more heterogeneous than PtFs with singular cells of substantially greater size (**Figure 3C**). Software-based quantification of cell size was conducted for PtFs and CAFs of all OPSCC donors. CAFs were significantly bigger in all but one patient (Pat.4) with a 1.64-fold difference in the mean cell size, which is driven by an increased number of moderately larger cells, and singular cells of substantially increased cell size. Thus, the proliferation rates of PtFs and CAFs differed slightly and CAFs showed reduced proliferation and increased doubling times over passages 1-7, and enhanced cell size, as compared to PtFs.

### Effects of PtFs and CAFs proliferation and migration of HNSCC cells

To explore potential effects of matched pairs of PtFs and CAFs, and of normal hOFs on the proliferation of HNSCC cell lines, normalized cell numbers of Cal27 and Cal33 cell lines (oral carcinoma), FaDu cells (hypopharynx), and Kyse30 cells (esophageous squamous carcinoma) were acquired following incubation with conditioned supernatants of PtF, CAF, and hOFs cultures. As a control, carcinoma cell lines were cultured in standard DMEM (Cal27, Cal33, and FaDu) or RPMI (Kyse30) in the absence of FCS, or in the presence of 10% FCS. As a further control, carcinoma cell lines were cultured in FGM for 72 hours. The resulting conditioned FGM served as a reference for PtFs and CAFs, and of normal hOFs conditioned medium.

**Figure 4:**
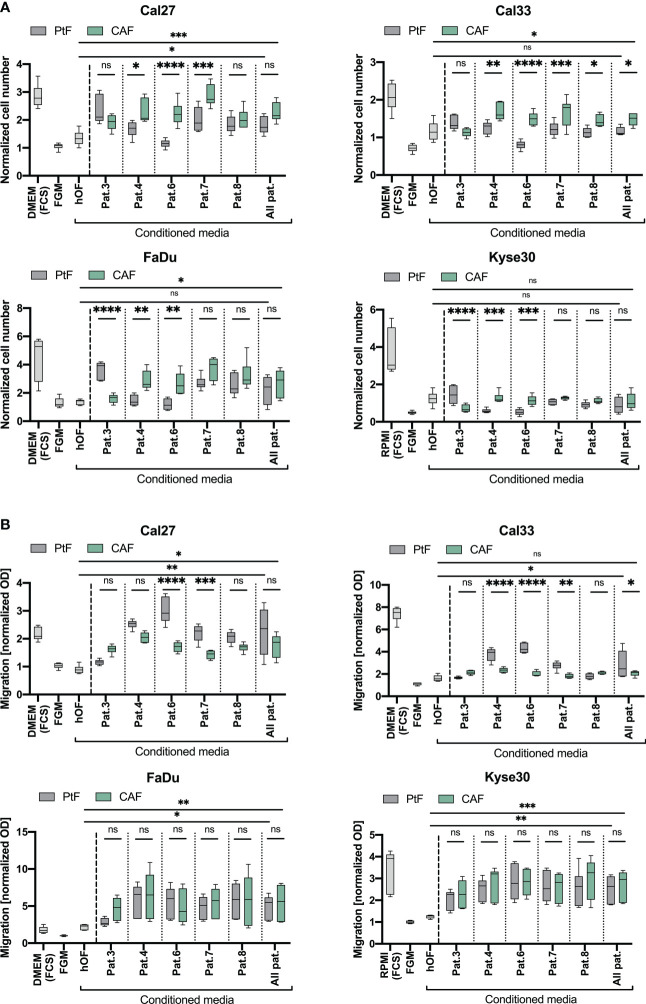
Effect of PtFs and CAFs on carcinoma cell line proliferation and migration. **(A)** Cal27, Cal33, FaDu and Kyse30 cell lines were cultured for 72 hours in human oral fibroblast (oHF)-, PtF-, or CAF-conditioned supernatant. Cell numbers are normalized to D0 and to DMEM/RPMI w/o FCS culture condition. Mean ± SD normalized cell numbers of controls (cell cultured in DMEM/RPMI with FCS, or in FGM) and treatment groups (cells cultured with hOF/PtF/CAF-conditioned FGM of individual patients) of n=3 independent experiments are shown. **(B)** Migration of Cal27, Cal33, FaDu and Kyse30 cells was measured using a Boyden chamber with FGM, DMEM/RPMI with FBS, and conditioned hOF/PtF/CAF-conditioned supernatant served as attractants in the lower chamber. Normalized OD values are plotted from n=3 individual experiments. P-values: ns > 0.05; * ≤0.05; ** ≤0.01; *** ≤0.001; **** ≤0.0001.

Cal27 and Cal33 showed significantly increased proliferation when cultured in conditioned medium from CAFs vs conditioned medium from PtFs, except Pat.3, where no differences were observed between PtFs and CAFs (**Figure 4A**). Supernatant of PtFs from Pat.3 and of CAFs of Pat.4 and 6 induced a significantly increased proliferation in FaDu and Kyse30 cells. Growth-promoting effects of CAF were likewise more pronounced in CAFs from Pat. 7 and 8 but did not reach statistical significance (**Figure 4A**). Overall, the growth-promoting effect of CAF conditioned medium was higher as compared to PtF conditioned medium. Except for Kyse30 cells, mean normalized cell numbers reached following treatment with CAF supernatants was significantly higher for all cell lines compared to treatment with hOF supernatants (**Figure 4A**). Differences in cell numbers treated with PtF or hOF supernatants were only seen for Cal27 cells. For all other cell lines, no differences were observed (**Figure 4A**).

To explore potential effects of supernatants of PtFs and CAFs, and of normal hOFs on tumor cell migration, a Boyden chamber assay was performed with Cal27, Cal33, FaDu, and Kyse30 cells following treatment with conditioned media. Treatment of Cal27 and Cal33 cell lines with PtF supernatants of Pat.4 (only Cal33), Pat.6, and Pat.7 led to significantly increased migratory potential, compared to treatment with their respective CAF supernatants (**Figure 4B**). Other treatment groups did not show any significant difference in treatment with CAF and PtF supernatants. In FaDu and Kyse30 cells, supernatants of PtFs and CAFs all induced an increased migration and showed no significant differences in matched pairs (**Figure 4B**). In all cell lines, CAF and PtF supernatants revealed significantly superior to hOF supernatants in inducing cell migration, except Cal33 in which CAF and hOF supernatant did not significantly differ (**Figure 4B**).

Hence, CAF and PtF supernatants promoted cell proliferation and migration of HNSCC and esophageal cancer cells.

### VEGF-A, TGFβ2, PDGF-BB, and BDNF secretion and angiogenic effects of CAFs and PtFs

**Figure 5:**
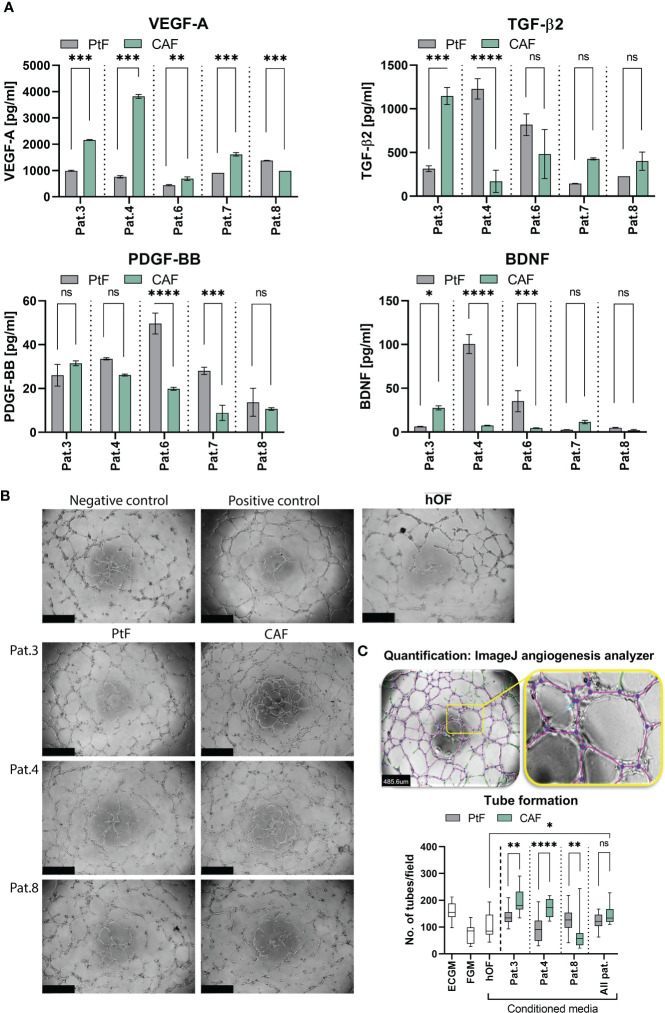
Growth factor secretion and effect of PtFs and CAFs on the angiogenic potential of HUVECs. **(A)** Secretion of VEGF-A, TGF-β2, PDGF-BB and BDNF of PtFs and CAFs was measured by ELISA in all patients (Pat.3, Pat.4, Pat.6, Pat.7 and Pat.8). Shown are mean ± SD values of each analyte from n=3 individual experiments. **(B)** Representative images showing angiogenic potential of HUVECs in ECGM (positive control, top right), and in FGM (negative control, top left). The lower panel shows representative images of tube formation in HUVECs when cultured in PtF/CAF-conditioned medium of Pat3, Pat.4 and Pat.8. Uper right picture shows HUVECs cultured in the presence of hOF supernatant. Images were captured after 8 hour treatment. **(C)** Analysis of tube formation ability in HUVECs (top panel) with purple lines depicting tubes between two connected junctions (blue dots) and green lines depicting unconnected segments. Number of connected tubes of HUVEC cultured in negative and positive controls, and with hOF/PtF/CAF-conditioned supernatants of individual patients are determined (bottom panel). Shown are mean ± SD values. P-values: ns > 0.05; * ≤0.05; ** ≤0.01; *** ≤0.001; **** ≤0.0001.

To address a potential impact of CAF and PtF populations on the functionality of cancer and endothelial cells, cytokine secretion of vascular endothelial growth factor A (VEGF-A), platelet-derived growth factor BB (PDGF-BB), transforming growth factor β2 (TGF-β2), and brain-derived neurotrophic factor (BDNF) by CAFs and PtFs was measured by ELISA. VEGF-A and PDGF-BB were chosen for their crucial involvement in (neo)-angiogenesis, TGF-β2 as an inducer of EMT that is an important contributor to tumor cell heterogeneity in HNSCC, and BDNF as a neurotrophin involved in perineural invasion of cancer cells. VEGF-A secretion was significantly higher in the CAF than in PtF populations across all patients, except Pat.8 where it was lower (**Figure 5A**, top left), whereas PDGF-BB was only significantly higher in the PtF populations in two patients (Pat.6 and Pat.7) (**Figure 5A**, lower left). TGF-β2 secretion in CAF/PtF matched pairs was heterogeneous across all patients. Only Pat.3 and Pat.4 showed significant differences in TGF-β2 secretion, with Pat.3 showing higher levels in CAF populations, and Pat.4 in PtF populations (**Figure 5A**, upper right). BDNF secretion levels were generally very low across all CAF and PtF populations, with Pat.4 and Pat.6 showing significantly higher BDNF secretion levels in their respective CAF populations, and Pat.3 slightly higher expression in PtF populations (**Figure 5A**, lower right).

Based on the overall high levels of VEGF-A secretion in both fibroblast subpopulations, a tube formation assay was performed with HUVECs to evaluate the angiogenic ability of CAFs and PtFs in comparison to hOF. Conditioned supernatants of Pat.3, Pat.4 and Pat.8 were chosen based on the highest differences in VEGF-A secretion between CAFs and PtFs (higher secretion by CAFs in Pat.3 and Pat.4; higher secretion by PtFs in Pat.8). Representative pictures of HUVECs treated as negative control in HUVEC-conditioned FGM, positive control in fresh ECGM with supplements, in hOF supernatant, and with conditioned media of CAF and PtF populations of three different patients, are shown in **Figure 5B**. Software-based analysis of the tube formation was conducted to define junctions and tubes. When comparing pro-angiogenic ability of fibroblast supernatants, the number of tubes formed by HUVECs treated with conditioned media of CAFs of Pat.3 and Pat.4 were significantly higher to than of PtFs. Oppositely, conditioned media of PtFs of Pat.8 induced significantly higher tube formation than its CAF counterpart (**Figure 5B**, right panel). Furthermore, conditioned media with the lowest VEGF-A levels (PtFs from Pat.3 and Pat4, CAFs from Pat.8) did not induce significant tube formation as compared to the negative control. Tube formation induced by CAF was significantly superior to hOF, whereas PtF supernatant showed no significant difference to hOF (**Figure 5B**). These results demonstrate an angiogenic capacity of CAFs and PtFs, which correlated with VEGF-A secretion as quantified in conditioned supernatants.

## Discussion

Understanding factors and regulatory mechanisms that influence tumor progression and metastasis formation is crucial to improve the prognosis of HNSCC patients. This is a complex task as numerous variables are involved, such as a high burden of genetic mutations, intratumor heterogeneity at the genome, transcriptome, and proteome level, and influences of the surrounding TME (29–33). The focus of this study was to isolate and characterize fibroblasts found within and from the vicinity of malignant tissue, and to address their potential effects on tumor progression. One major rationale for this approach lies in the frequent development of local recurrences that are defined as carcinomas arising within a time period of three years post treatment and at a distance of max. two cm from the initial primary tumor (34). Local recurrences appear in 10 – 30% of cases with an R0 resection and hence arise despite a lack of microscopically detectable residual tumor cells in the resection margin upon histopathologic intraoperative examination. It is generally accepted that minimal residual disease (MRD) cells and fields of premalignant tissue are major sources for these recurrences. Macroscopically normal epithelium in the periphery of primary HNSCC may not only contain premalignant epithelial cells, but also stromal cells that contribute to tumor progression as part of the TME. Thus, the existence and function of PtFs is of particular interest in HNSCC owing to the substantial impact of field cancerization in these tumors. Accordingly, we have isolated fibroblasts from HNSCC patients with a differential spatial distribution within the tumor and in proximity of resection margins. We have explored their biological characteristics and effects of secreted soluble factors on carcinoma cells of the upper aerodigestive tract and compared them to normal hOFs. PtFs were isolated from tissue beyond microscopically clear resection margins as proven by intraoperative frozen section analysis. Although the tissue appeared macroscopically normal, it may nonetheless contain pre-malignant fields (**Figure 1A**). PtFs expressed classical fibroblast markers (positive markers: CD90, CD73, CD105, α-SMA, Vimentin, S100A4; negative markers: CD45, CD34, CD31, E-cadherin, Pan-cytokeratin) with apparent differences to neither CAFs nor hOFs.

CAFs assume key roles in the TME that can directly or indirectly affect the development of tumor cells through secretion of growth factors and cytokines, ECM production, and *via* direct cell-cell interactions in numerous carcinomas including HNSCC (8, 35, 36). Soluble growth factors secreted by CAFs, such as VEGF-A can promote neo-angiogenesis and, thereby, sustain tumor growth and dissemination (37). CAFs further secrete TGF-β and IL-6, which affect immune crosstalk and interfere with immune cell function to allow tumor cells to escape host immunity. CAF-derived TGF-β also promotes a partial differentiation of tumor cells within HNSCC along the EMT (partial EMT, pEMT), which is associated with the presence of lymph node metastasis (38, 39) and poorer clinical outcome (40). In analogy to carcinoma cells, phenotypic and transcriptomic analyses have demonstrated that CAFs can be divided into various subtypes, with each subtype exhibiting unique characteristics and functions (8, 24, 41, 42). In pancreatic ductal adenocarcinoma (PDAC), a subpopulation of CAFs located in the direct proximity of tumor cells and characterized by high expression of α-SMA produces desmoplastic stroma (41, 43). Production of collagen-rich, dense desmoplastic stroma promotes neoplastic growth, metastatic spread, and generates a natural barrier for therapeutic agents. A second CAF subpopulation located more distant to pancreatic cancer cells did not express elevated α-SMA levels and was characterized by the production of pro-inflammatory cytokines, such as IL-6. In HNSCC, CAF subtypes have been described based on single-cell and bulk RNA sequencing data. Zhang et al. have reported that CAFs in HNSCC patients can be divided into eight distinct clusters based on the total of n=1,423 single fibroblasts determined in single-cell analysis (24). The expression of marker genes for these eight CAF clusters revealed that seven clusters were enhanced in tumor vs. normal tissue, and that three clusters were associated with reduced overall survival (OS). Similarly to PDAC, one HNSCC-derived CAF cluster was associated with enhanced α-SMA expression and myofibroblast characteristics. Two other clusters with prognostic significance were enriched in EMT genes and in genes associated with antigen presentation. Based on a large cohort study (n=587 HNSCC samples) investigating the prognostic significance of myofibroblastic CAFs it was found that patients with moderate/high levels of stromal α-SMA had substantially higher disease-specific mortality than low/negative patients. This indicates that the disease-specific mortality of patients is correlated with CAFs harboring various amounts of α-SMA expression (44) The analysis of α-SMA in HNSCC-derived PtFs and CAFs in our study did not disclose any significant differences across all patients on average, but two patients showed higher α-SMA expression in CAFs compared to PtFs (both HPV-neg.), and one patient showed higher levels in PtFs compared to CAFs (HPV-pos.), which may hint at singular CAFs with differing functionality. Although an association with the HPV-status was seen, the small sample size does not allow to draw firm conclusions.

Regarding doubling times, CAFs generally showed increasing doubling times compared to their matched PtFs (**Figure 3**). Proliferation rates were variable for CAF/PtF pairs across all patients. The PtF populations showed higher proliferation rates in two patients (Pat.6 and Pat.7), and the CAF populations showed higher proliferation rates compared to PtF populations in two other patients (Pat.3 and Pat.8). These results suggested a certain degree of inter-patient heterogeneity between PtFs and CAFs, and showed reduced proliferation compared to normal oHFs. Furthermore, CAFs exhibited increased heterogeneity in terms of cell size compared to PtFs and contained singular large cells (**Figure 3**). The different characteristics of CAFs and PtFs may be the consequence of their different spatial location. For example, tumor cell apoptosis within the tumor leads to a less organized and more loose ECM structure and, thus, provides more space for them to grow (30, 45). The central area of the tumor often lacks oxygen and nutrients, which may cause CAFs to adopt a higher doubling time to adapt to hypoxia and scarce nutrients (46). Hypoxia may also cause chemotherapy resistance and changes in TMEs (47, 48).

We further concentrated on soluble factors secreted by CAFs, PtFs, and hOFs on carcinoma cells of the upper aerodigestive tract. Soluble factors were chosen to focus on potential paracrine effects of PtFs on carcinoma cells rather than cell-cell contact-based influences of fibroblasts. Conditioned media of CAFs promoted proliferation of Cal27, Cal33, and FaDu carcinoma cells, which was significantly higher than effects of hOFs (**Figure 4**). A proliferation-inducing capacity of CAFs is in line with the general view of growth-promoting effects of tumor-resident CAFs (13, 49, 50). Effects of CAFs and PtFs on cancer cell migration was assessed by *in vitro* measurements. Where significant differences were observed in matched samples (i.e. in Cal27 and Cal33), PtF-conditioned supernatant showed a higher capacity to induce tumor cell migration, compared to matched CAFs. Furthermore, PtF- and CAF-derived supernatants generally induced higher migration than hOF-derived soluble factors with significant differences in all cell lines except for CAF supernatant on Cal33 cells. Such increased capacity to induce tumor cell migration by PtFs may support the detachment and migration/invasion of single tumor cells or small clusters into macroscopically normal tissue and foster MRD.

Across four out of five patients, VEGF-A secretion levels of CAFs were higher compared to PtFs (**Figure 5**). This is consistent with CAFs residing in a potentially hypoxic environment within the tumor. Higher VEGF-A levels may allow the formation of new blood vessels within the tumor to support growth of the tumor (51, 52). This notion is supported by tube formation assays, which confirmed an angiogenic capacity that correlated with VEGF-A expression (**Figure 5**). However, it must be noted that despite being enhanced in CAF-conditioned supernatant, VEGF-A levels of both CAFs and PtFs were generally high. Thus, conditioned media of PtF also displayed pro-angiogenic capacity and may support the neo-vascularization of locally recurring carcinomas. However, unlike CAFs the difference in angiogenic activity between PtFs and hOF was not significant.

During the process of fibroblast activation to CAFs, many growth factors are involved, one of them being PDGF-BB. This growth factor can not only promote angiogenesis but also activate fibroblasts to further transform to CAFs (9). PtFs show higher PDGF-BB secretion levels compared to CAFs, which may indicate that fibroblast subtypes can promote differently angiogenesis and tumor growth. These findings also suggest that PtFs may potentially have a stronger ability to transform by-stander fibroblasts near the tumor to CAFs to additionally support tumor progression (53, 54). PtFs and CAFs can both secrete TGF-β2 and BDNF, which can promote EMT, metastasis, invasion, and therapy resistance (55–61). However, it must be noted that while TGF-β2 levels were high, BDNF concentration were comparably low.

A limitation of the present study is seen in the small sample size of n = 5 pairs of CAFs and PtFs. In combination with the implementation of HPV-negative and -positive patients, differences in antigen expression, proliferation, and paracrine effects within and across patients must be judged with caution. Studies implementing larger numbers of patients and cognate pairs of CAFs and PtFs would therefore complement the present study and help discern differences and similarities between fibroblast types with more statistical rigor. A further limitation that should be noted is the choice of an *in vitro* experimental setup to study effects of CAFs and PtFs on carcinoma cell lines. While this *in vitro* model does not allow to draw conclusions on potential effects in the context of a whole organism, it did allow to address specific questions (i.e. the direct effects of fibroblast-derived soluble factors on carcinoma cells) without secondary effects stemming from the complexity of e.g. a small animal model.

In summary, we have successfully isolated and cultured peritumoral fibroblasts present in the vicinity of primary tumors. Characteristics and biological functions of PtFs and CAFs were compared in matched pairs from five HNSCC patients. Our results show that although PtFs originate from macroscopically normal tissue surrounding the tumors, they induced carcinoma cell migration more strongly than normal hOFs. The fact that we isolated and cultured fibroblasts from mucosa beyond clear resection margins can further be seen as a cause for the far-reaching effects of malignant growth and/or field cancerization, most notably the high locoregional recurrence rates of 9-26% of OPSCC patients (62). Moving forward, more research is needed to study the influence of spatial distribution of CAFs and PtFs on tumor progression to provide more information on CAF- and PtF-induced tumor growth and invasion mechanisms.

## Data availability statement

The original contributions presented in the study are included in the article/**Supplementary Material**. Further inquiries can be directed to the corresponding author.

## Ethics statement

The studies involving human participants were reviewed and approved by Ethikkommission Medizinische Fakultät LMU München; Ethics number 18-446. The patients/participants provided their written informed consent to participate in this study.

## Author contributions

JZ, SS-Z, GK, SD have conducted experiments and analysed data. JZ, SS-Z, VK, OG have generated figures. CW and FK have performed pathological analyses. MC, OG, FH, PB, VK have analysed and interpreted data, and have helped writing and correcting the manuscript. FH, OG, PB, VK coordinated the study. JZ, OG, VK wrote the initial manuscript draft. All authors contributed to the article and approved the submitted version.

## Funding

Jiefu Zhou was sponsored by the Chinese Scholarship Council (CSC) for a three-year doctoral study at the Ludwig-Maximilians-University (LMU).

## Conflict of interest

The authors declare that the research was conducted in the absence of any commercial or financial relationships that could be construed as a potential conflict of interest.

## Publisher’s note

All claims expressed in this article are solely those of the authors and do not necessarily represent those of their affiliated organizations, or those of the publisher, the editors and the reviewers. Any product that may be evaluated in this article, or claim that may be made by its manufacturer, is not guaranteed or endorsed by the publisher.

## Supplementary material

Supplementary Figure 1 Immunocytochemistry (ICC)-based characterization of oHFs. Expression of epithelial markers (E-cadherin, pan-cytokeratin (Pan-CK)), endothelial marker (CD31), and the fibroblast markers (α-SMA, Vimentin, S100A4) in oHFs was assessed through ICC staining and counter staining with hematoxylin-eosin in early passages (< 10). Representative images are shown.

Supplementary Table 1 List of BioLegend antibodies and isotype controls used for flow cytometry
