# Supplementary figures and images for: Isolation and characterization of head and neck cancer-derived peritumoral and cancer-associated fibroblasts
Source: Front Oncol. 2022 Dec 5;12:984138. doi: 10.3389/fonc.2022.984138 (PMC9760815; doi:10.3389/fonc.2022.984138)

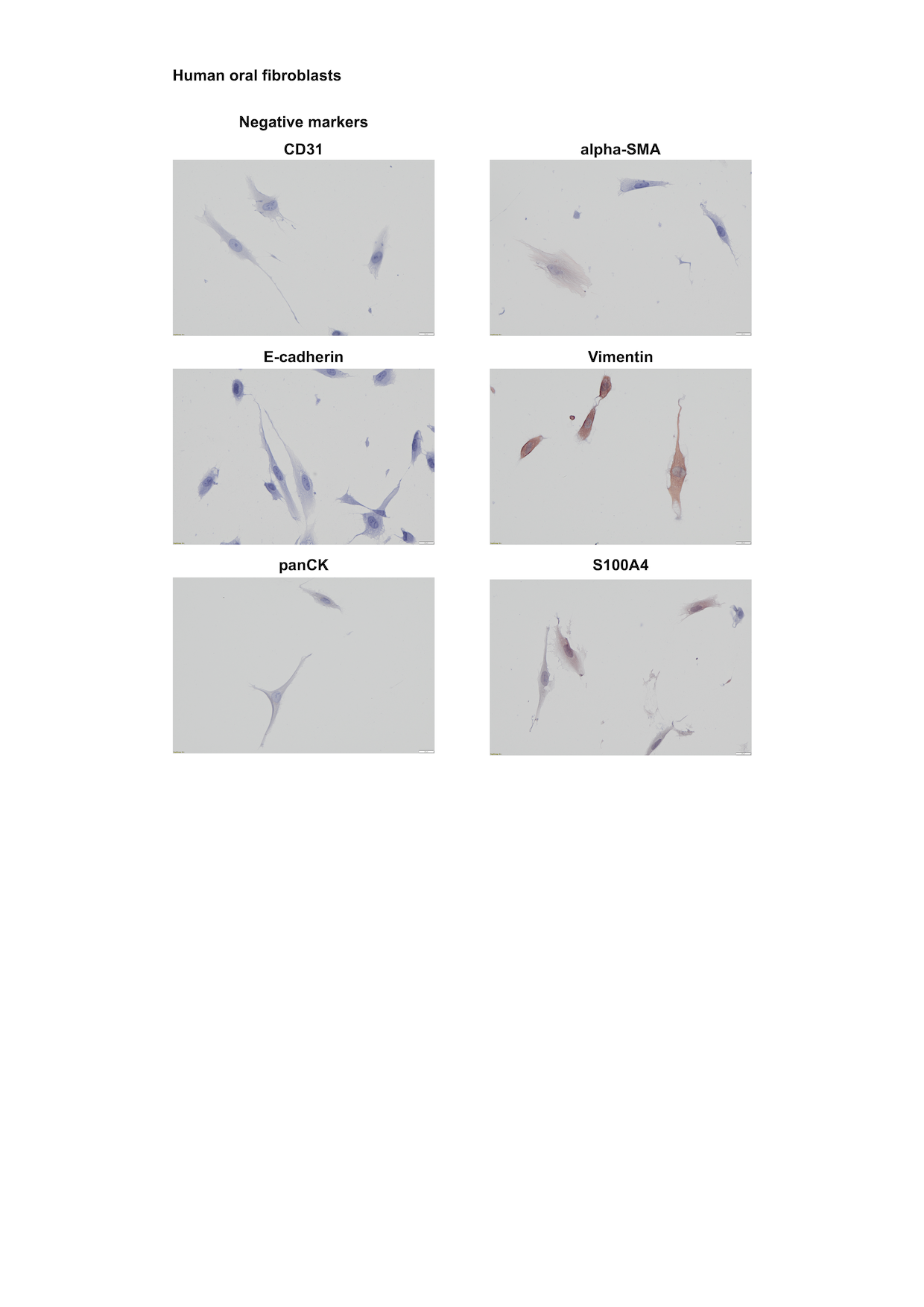

Supplement: Supplementary Figure 1 — Immunocytochemistry (ICC)-based characterization of oHFs. Expression of epithelial markers (E-cadherin, pan-cytokeratin (Pan-CK)), endothelial marker (CD31), and the fibroblast markers (α-SMA, Vimentin, S100A4) in oHFs was assessed through ICC staining and counter staining with hematoxylin-eosin in early passages (< 10). Representative images are shown. [file Image_1.tif]
