# Supplementary material for: Isolation and characterization of head and neck cancer-derived peritumoral and cancer-associated fibroblasts
Source: Front Oncol. 2022 Dec 5;12:984138. doi: 10.3389/fonc.2022.984138 (PMC9760815; doi:10.3389/fonc.2022.984138)
Supplement: Supplementary Table 1 — List of BioLegend antibodies and isotype controls used for flow cytometry [file Table_1.docx]

**Supplementary Table 1**: List of BioLegend antibodies and isotype controls used for flow cytometry

| **Antibody target** | **Fluorophore conjugate** | **Catalog no.** |
| --- | --- | --- |
| Anti-human CD90 (Thy1) | APC | #328113 |
| Anti-human CD73 (Ecto-5’-nucleotidase) | APC | #344005 |
| Anti-human CD105 | Alexa Fluor® 488 | #323209 |
| Anti-human CD31 | APC | #303115 |
| Anti-human CD34 | Alexa Fluor® 488 | #343517 |
| Anti-human CD45 | Alexa Fluor® 488 | #304019 |
| Mouse IgG1 isotype control | APC | #400119 |
| Mouse IgG1 𝜅 isotype control | Alexa Fluor® 488 | #400132 |
